# Mesial Temporal Lobe Epilepsy (MTLE) Drug-Refractoriness Is Associated With P2X7 Receptors Overexpression in the Human Hippocampus and Temporal Neocortex and May Be Predicted by Low Circulating Levels of miR-22

**DOI:** 10.3389/fncel.2022.910662

**Published:** 2022-07-07

**Authors:** Bárbara Guerra Leal, Aurora Barros-Barbosa, Fátima Ferreirinha, João Chaves, Rui Rangel, Agostinho Santos, Cláudia Carvalho, Ricardo Martins-Ferreira, Raquel Samões, Joel Freitas, João Lopes, João Ramalheira, Maria Graça Lobo, António Martins da Silva, Paulo P. Costa, Paulo Correia-de-Sá

**Affiliations:** ^1^Unit for Multidisciplinary Research in Biomedicine (UMIB), Instituto de Ciências Biomédicas Abel Salazar—Universidade do Porto (ICBAS-UP), Porto, Portugal; ^2^Immunogenetics Laboratory, Molecular Pathology and Immunology Department, ICBAS-UP, Porto, Portugal; ^3^Laboratory for Integrative and Translational Research in Population Health (ITR), Porto, Portugal; ^4^Laboratório de Farmacologia e Neurobiologia—Center for Drug Discovery and Innovative Medicines (MedInUP), ICBAS-UP, Porto, Portugal; ^5^Serviço de Neurologia, Hospital de Santo António—Centro Hospitalar e Universitário do Porto (HSA-CHUP), Porto, Portugal; ^6^Serviço de Neurocirurgia, HSA-CHUP, Porto, Portugal; ^7^Serviço de Patologia Forense, Instituto Nacional de Medicina Legal e Ciências Forenses—Delegação do Norte (INMLCF-DN), Porto, Portugal; ^8^Serviço de Neurofisiologia, HSA-CHUP, Porto, Portugal; ^9^Departamento de Genética, Instituto Nacional de Saúde Dr. Ricardo Jorge, Porto, Portugal

**Keywords:** mesotemporal lobe epilepsy, hippocampus, microRNAs, P2X7 purinoceptor, miR-22, refractory epilepsy

## Abstract

**Objective**: ATP-gated ionotropic P2X7 receptors (P2X7R) actively participate in epilepsy and other neurological disorders. Neocortical nerve terminals of patients with Mesial Temporal Lobe Epilepsy with Hippocampal Sclerosis (MTLE-HS) express higher P2X7R amounts. Overexpression of P2X7R bolsters ATP signals during seizures resulting in glial cell activation, cytokines production, and GABAergic rundown with unrestrained glutamatergic excitation. In a mouse model of status epilepticus, increased expression of P2X7R has been associated with the down-modulation of the non-coding micro RNA, miR-22. MiR levels are stable in biological fluids and normally reflect remote tissue production making them ideal disease biomarkers. Here, we compared P2X7R and miR-22 expression in epileptic brains and in the serum of patients with MTLE-HS, respectively.

**Methods**: Quantitative RT-PCR was used to evaluate the expression of P2X7R in the hippocampus and anterior temporal lobe of 23 patients with MTLE-HS and 10 cadaveric controls. Confocal microscopy and Western blot analysis were performed to assess P2X7R protein amounts. MiR-22 expression was evaluated in cell-free sera of 40 MTLE-HS patients and 48 healthy controls.

**Results**: Nerve terminals of the hippocampus and neocortical temporal lobe of MTLE-HS patients overexpress (*p* < 0.05) an 85 kDa P2X7R protein whereas the normally occurring 67 kDa receptor protein dominates in the brain of the cadaveric controls. Contrariwise, miR-22 serum levels are diminished (*p* < 0.001) in MTLE-HS patients compared to age-matched control blood donors, a situation that is more evident in patients requiring multiple (>3) anti-epileptic drug (AED) regimens.

**Conclusion**: Data show that there is an inverse relationship between miR-22 serum levels and P2X7R expression in the hippocampus and neocortex of MTLE-HS patients, which implies that measuring serum miR-22 may be a clinical surrogate of P2X7R brain expression in the MTLE-HS. Moreover, the high area under the ROC curve (0.777; 95% CI 0.629–0.925; *p* = 0.001) suggests that low miR-22 serum levels may be a sensitive predictor of poor response to AEDs among MTLE-HS patients. Results also anticipate that targeting the miR-22/P2X7R axis may be a good strategy to develop newer AEDs.

## Introduction

Mesial Temporal Lobe Epilepsy with Hippocampal Sclerosis (MTLE-HS) is the most frequent partial epilepsy in adults. Patients often present a history of Febrile Seizures (FS) in childhood and more than 80% are refractory to first-line anti-epileptic drugs (AEDs) requiring multiple drug regimens with limited success. In such cases, surgical ablation of the hippocampus and amygdala is the last resource to control epileptic seizures, yet this procedure may have devastating effects on patients’ quality of life and constitutes a significant burden for national health care systems. Notwithstanding this, seizure recurrence may occur 10–18 years after surgery in 47% and 38% of the patients, respectively (Jeha et al., 2007; Hemb et al., 2013), and these patients remain with unmet clinical needs. While understanding the epileptogenic process is paramount to the development of newer AEDs, this task has been hampered because the mechanism(s) leading to MTLE-HS remain largely unknown.

Mounting evidence suggests a role for microRNAs (miRs) in the epileptogenic process (Henshall et al., 2016). These small non-coding RNA molecules function as post-transcriptional regulators of gene expression controlling biological processes, such as immune responses and neurotransmission. Different miR expression profiles have been described concerning epilepsy, with several miRs being up or downregulated after epileptic seizures, both in human patients and in animal models (Wang et al., 2015; Henshall et al., 2016). Interestingly, miRs are quite stable in biological fluids, such as plasma or serum, and normally reflect remote tissue production (Turchinovich et al., 2012), so circulating miRs have been proposed as promising novel biomarkers for diagnosis, prognosis and/or optimization of the anti-epileptic treatment (Wang et al., 2015; Henshall et al., 2016). In animal models, targeting specific miR molecules is associated with seizure reduction (Henshall, 2013). Circulating miR-22 is downregulated in an animal model of *status epilepticus* (Jimenez-Mateos et al., 2015). MiR-22 also displays a neuroprotective role in regulating neuronal death and apoptosis in a model of traumatic brain injury (Ma et al., 2016). It is also involved in the regulation of neuronal excitability, neuroinflammation, and aberrant neurogenesis, which are mechanisms tightly linked to ATP-mediated dangerous actions *via* P2X7 purinoceptors (P2X7R) activation (Jimenez-Mateos et al., 2015).

P2X7 purinoceptors are ligand-gated ion channels exhibiting low affinity for ATP, whose activation is only possible under conditions that favor extracellular ATP accumulation roughly to the millimolar concentration range (Rassendren et al., 1997; Sperlagh and Illes, 2014). Sustained extracellular ATP accumulation and subsequent P2X7 receptors activation are more likely during stressful conditions, such as a consequence of brain damage, hypoxia, and excessive neuronal activity during epileptic seizures. Yet, transient activation of this receptor may also be functionally relevant under physiological conditions, such as during synaptic plasticity phenomena triggered by high-frequency stimuli inherent to learning and memory processes (Campos et al., 2014). Once activated, the P2X7R behaves as a non-desensitizing cation channel involved in the long-lasting influx of Na^+^ and Ca^2+^ and in the efflux of K^+^, depending on ionic concentration gradients (Rassendren et al., 1997; Skaper, 2011). Prolonged P2X7R activation may lead to the formation of a reversible plasma membrane pore permeable to hydrophilic molecules up to 900 Da (Surprenant et al., 1996; Noronha-Matos et al., 2014). Using both human and animal preparations of the central nervous system (CNS), it has been demonstrated that the P2X7R is expressed in neurons, astrocytes, and microglia having pleiotropic effects modulating neuron-glia interaction, host defense, and neuroinflammation (Sperlagh and Illes, 2014). In microglial cells, the P2X7R has a trophic function modulating their activation and proliferation (Monif et al., 2009), which leads to the expression of pro-inflammatory cytokines, Il-1β and TNF-α, and reactive oxygen species (Ferrari et al., 1997; Skaper et al., 2010; Choi et al., 2012). Through this action, P2X7R may modulate neuronal cell death (Kanellopoulos and Delarasse, 2019). The presence of the P2X7R in pre-synaptic nerve terminals and astrocytes explains its role in GABA and glutamate release (Marcoli et al., 2008). Our group has shown that P2X7R is overexpressed in neocortical nerve terminals of drug-resistant epileptic patients leading to down-modulation of GABA and glutamate uptake, which endures GABA signaling, increases GABAergic rundown, and, thereby, unbalances glutamatergic neuroexcitation (Barros-Barbosa et al., 2016). The presence of P2X7R in nerve terminals is controversial probably reflecting region-specific or pathology-associated neuronal expression. Upregulation of the P2X7R expression has also been verified in the hippocampus and cortex of animal models (Engel et al., 2012a; Jimenez-Pacheco et al., 2013, 2016; Barros-Barbosa et al., 2015, 2018; Morgan et al., 2020) contributing to resistance to pharmacotherapy during status epilepticus (Beamer et al., 2021). The neocortex of human patients with TLE also overexpress the P2X7R (Jimenez-Pacheco et al., 2013, 2016; Barros-Barbosa et al., 2016), but there is a gap in our knowledge concerning the location of this in the human hippocampus.

Recently, it was demonstrated that the P2X7R plasma levels were higher in rats with TLE than in control animals suggesting that it might have a putative role as a biomarker for the diagnosis and therapeutic follow-up of epilepsy (Conte et al., 2021). Interestingly, P2X7R antagonists reduce the number and duration of spontaneous seizures and gliosis, which effects are maintained beyond treatment cessation (Jimenez-Pacheco et al., 2013, 2016). The association between P2X7R antagonism and seizure suppression was confirmed by other research groups (Mesuret et al., 2014; Amhaoul et al., 2016; Amorim et al., 2017). Considering that increased expression of the P2X7R has been associated with down-modulation of the non-coding microRNA, miR-22, at the post-transcriptional level in a mouse model of status epilepticus (Jimenez-Mateos et al., 2015; Engel et al., 2017), we now set to compare the expression of P2X7R and miR-22 in epileptic brains and sera, respectively, of MTLE-HS patients.

## Material and Methods

### Brain Samples From Epileptic Patients and Cadaveric Controls

Resected fresh human brain samples were obtained from 23 MTLE-HS patients (13F/10M, 39.6 ± 9.8 years old; see Table 1) who underwent epilepsy surgical treatment (selective amygdalohippocampectomy or anterior temporal lobectomy) at the Neurosurgery Department of Hospital Santo António—Centro Hospitalar e Universitário do Porto (HSA-CHUP). The decision for surgery was taken by HSA-CHUP multidisciplinary epilepsy team incorporating neurologists, neurosurgeons, neuroradiologists, neurophysiologists, and neuropsychologists. All patients were resistant to maximal doses of two to four conventional AEDs used for more than two consecutive years (Table 1). Pre-surgical assessment was discussed by the team analyzing the results of brain MRI, prolonged video-EEG recording, ictal and interictal SPECT, neuropsychological assessment, and functional brain MRI, in order to determine the suitability of patients for surgical intervention.The antiepileptic drug therapy was not suspended before surgery; multi-AED combination regimens included 15 different drugs, most often comprising carbamazepine (*n* = 12), clonazepam (*n* = 11), levetiracetam (*n* = 8), topiramate (*n* = 6), valproic acid (*n* = 6) and oxcarbazepine (*n* = 5). The postoperative outcome was evaluated 7 years after surgery using the Engel Epilepsy Surgery Outcome Scale. The majority of patients were seizure-free (level I) or presented rare disability seizures (level II); only one patient showed no improvement after surgery (level IV).

**Table 1:** Clinical and demographic data from MTLE-HS patients submitted to surgery.

**Clinical/demographic data**	**MTLE-HS (n total = 23)**
F/M	13 /10
Age at surgery ± SD, years (range)	39.6 ± 9.8 (24–60)
Age of onset ± SD, years (range)	10.3 ± 6.8 (1–28)
Disease mean duration ± SD, years (range)	29.3 ± 9.0 (10–49)
Number of antiepileptic drugs at surgery (AEDs = 2 / 3 / 4)	8 / 11 / 4
Hippocampal Sclerosis (Left /Right)	15 / 8
Febrile seizures antecedents (Yes / No)	15 / 8
Engel classification (I / II / III / IV)	16 / 2 / 4 / 1

*AEDs, antiepileptic drugs; F, female; M, male; SD, standard deviation*.

Surgical specimens of the hippocampus and anterior temporal lobe (neocortex) were collected. A complete coronal slice of 0.5 cm thickness was removed 3 cm posterior to the tip of the temporal pole. Samples were recovered in ice-cold artificial cerebrospinal fluid (CSF; 10 mM glucose, 124 mM NaCl, 3 mM KCl, 1 mM MgCl_2_, 1.2 mM NaH_2_PO_4_, 26 mM NaHCO_3_, 2 mM CaCl_2_, pH = 7.40) and immediately cryopreserved in liquid nitrogen. The amount of tissue removed did not differ from the strictly necessary for successful surgical practice. Serum samples from the peripheral blood of nine out of the 23 MTLE-HS patients (4 F/5 M) were obtained for circulating miR-22 expression quantification (see below); three of these samples were collected at the time of surgery and the remaining were collected at the nearest blood test control. This study and all its procedures were approved by the Ethics Committees of HSA-CHUP and ICBAS-UP. All patients gave written informed consent, and the investigation conforms to the principles outlined in *The Code of Ethics of the World Medical Association* (Declaration of Helsinki).

Control brain samples were obtained using an identical procedure from 10 human cadavers (8 M/2 F, 67.0 ± 10.9 years old) with no previous history of neurologic disease, which were submitted to forensic autopsies performed within 4–7 h after post-mortem that corresponds to the tissue viability window for functional assays (see Barros-Barbosa et al., 2016). The indiscriminate occurrence of forensic autopsies to individuals meeting the inclusion criteria and the short post-mortem time-frame required to obtain good quality brain samples for functional and molecular assays are limitations difficult to overcome concerning sample size and matching of age/gender with the patients’ group. Brain samples were discarded if post-collection assessment revealed active infectious or neoplastic diseases, chronic inflammatory conditions, neurologic poisoning, and/or past history of alcoholism and drug addiction. Brain samples were made available by the Instituto Nacional de Medicina Legal e Ciências Forenses—Delegação do Norte (INMLCF-DN), according to Decree-Law 274/99, of 22 July, published in Diário da República—1st SERIE A, No. 169, of 22-07-1999, Page 4522, regarding the regulation on the ethical use of human cadaveric tissue for research. After collection, brain tissue was kept in cold artificial CSF (see above) until use.

### Quantification of the P2X7R Gene Expression in Human Brain Tissue Samples

RNA was isolated from the fresh brain tissue, using the commercially available extraction kit RNeasy^®^ Blood/Tissue kit (Qiagen, Germany) according to the manufacturer’s instructions. RNA quantification and purity were evaluated using a Nanodrop Spectrophotometer (Thermo Scientific). Samples with an absorbance ratio at 260/280 nm between 1.5 and 2.0 were considered acceptable. RNA degradation was not assessed but according to previous reports, it remains stable for at least 36 h post-mortem (White et al., 2018). cDNA was synthesised with an available commercial kit (Nzy First-Strand cDNA Sunthesis Kit) in a BiometraAlfagene thermocycler, according to the manufacturer’s instructions. P2X7R (hs0017521_m1) and the reference gene Ubiquitin C (UBC; hs00824723_m1) expression were quantified by Real Time PCR with specific primers and probes (Taqman^®^ Kits, Applied Biosystems, USA) and a NzySpeedy qPCR mastermix (Nzytech, Portugal) in Corbett Rotor Gene 600 Real Time Thermocycler machine (Corbett Research, UK). UBC gene was chosen as the reference gene since its expression showed relatively low variability in expression levels in the regions studied (Trabzuni et al., 2011). Each reaction was performed in triplicate and the average Ct value was used in the analysis. The relative expression was calculated using the 2^−ΔΔCT^ method.

### Immunolocalization of P2X7R in the Human Hippocampus by Confocal Microscopy

Coronal sections of the hippocampus were fixed in 4% paraformaldehyde, cryopreserved in 30% sucrose, and stored in a tissue freezing medium at −80°C. Free-floating 30-μm hippocampal sections were incubated for 1 h, with blocking buffer (fetal bovine serum 10%, BSA 1%, triton X-100 0.5%, NaN_3_ 0.05%) and incubated overnight with the primary antibodies: rabbit anti-P2X7R (1:50, Alomone #APR-004, Jerusalem, Israel), goat anti-synaptic vesicle-associated membrane protein 1 (VAMP-1; 1:20, R&D Systems #AF4828, Minneapolis, MN, USA), and mouse anti-glial fibrillary acidic protein (GFAP, 1:200, Chemicon #MAB360, Temecula, CA, USA). Sections were rinsed and incubated for 2 h with species-specific secondary antibodies (donkey anti-rabbit Alexa Fluor 488, goat anti-mouse Alexa Fluor 633, and donkey anti-goat Alexa Fluor 633; Molecular Probes, Eugene, OR, USA). After mounting, sections were observed and analyzed with a laser scanning confocal microscope (Olympus FV1000, Tokyo, Japan). Co-localization was assessed by calculating the staining overlap and the Pearson’s coefficient (ρ) for each confocal micrograph using the Olympus Fluoview 4.2 Software (Olympus FV1000, Tokyo, Japan), as previously described (Barros-Barbosa et al., 2016).

### Immunoblot Analysis of the P2X7R Protein Amounts in Isolated Nerve Terminals of the Human Hippocampus

Total membrane lysates and nerve terminals of the hippocampus were isolated for immunoblot quantification of P2X7R protein amounts, as described previously (Bancila et al., 2009; see also Barros-Barbosa et al., 2016). Briefly, fragments of the hippocampus were gently homogenized in cold oxygenated (95% O_2_/5% CO_2_) Krebs solution (in mM: glucose 5.5, NaCl 136, KCl 3, MgCl_2_ 1.2, Na_2_HPO_4_ 1.2, NaHCO_3_ 16.2, CaCl_2_ 0.5, pH 7.40). The resulting homogenates were filtered through a nylon filter (mesh size 100 μm) to isolate nerve terminal membranes. The filtrate was left to sit until pellet formation, which was resuspended into Krebs solution and left at room temperature. The protein concentration was determined by the bicinchoninic acid method (Pierce, Thermo Scientific, Rockford, IL, USA) and was adjusted to 6.25 mg protein/mL. Membrane lysates and isolated nerve terminals were then homogenized in radioimmunoprecipitation assay buffer [Tris-HCl 25 mM (pH 7.6), NaCl 150 mM, sodium deoxycholate 1%, Triton-X-100 1%, SDS 0.1%, EDTA 5 mM, and protease inhibitors]. The samples were solubilized in SDS buffer [Tris-HCl 125 mM (pH 6.8), SDS 4%, bromophenol blue 0.005%, glycerol 20%, and 2-mercaptoethanol 5%], subjected to electrophoresis in SDS-polyacrylamide gels, and electrotransferred onto polyvinylidene difluoride (PVDF) membranes (Merck Millipore, Darmstadt, Germany). The membranes were blocked in Tris-buffered saline [in mM: Tris-HCl 10 (pH 7.6), NaCl 150] containing Tween-20 0.05% and BSA 5% and incubated with the primary antibodies: mouse anti-GFAP (1:500, Chemicon, Temecula, CA, USA), mouse anti-synaptophysin (1:750, Chemicon, Temecula, CA), and rabbit anti-P2X7R (1:200; Alomone #APR-004, Jerusalem, Israel). Then, the membranes were washed and incubated with horseradish peroxidase–conjugated secondary antibody. For normalization purposes, the membranes were incubated with rabbit anti-β-actin antibody (1:1,000; Abcam, Cambridge, United Kingdom) or mouse anti-glyceraldehyde 3-phosphate dehydrogenase (GAPDH) antibody (1:200; Santa Cruz Biotechnology, Dallas, TX, USA) following the procedures described earlier. The antigen–antibody complexes were visualized using the ChemiDoc MP imaging system (Bio-Rad Laboratories, Hercules, CA, USA). Gel band image densities were quantified using Image J (National Institutes of Health, Bethesda, MD, USA). To test for specificity of the bands corresponding to P2X7R, the anti-P2X7R antibody was pre-adsorbed with a control peptide antigen corresponding to the amino acid residues 576–595 of the intracellular C-terminus of the P2X7R.

### Quantification of the miR-22 Expression in the Human Serum

Serum samples were obtained from peripheral blood of 40 MTLE-HS patients (23 F/17 M, 43.0 ± 12.2 years old; 30 of them refractory to treatment; see Table 2) and 48 age-matched controls (28 F/20 M, 42.0 ± 10.8) without known neurological diseases. Patients were followed up at the Epilepsy Outpatient Clinic of the HSA-CHUP. All patients had MTLE-HS diagnosis based on clinical and electrophysiological studies (EEG and/or video-EEG monitoring) and on brain MRI (minimum 1.5 T) features. The definition of HS was based on brain MRI findings criteria which comprised atrophy, T2 hyperintensity signal, and altered internal structure on one or both hippocampi associated or not with other imaging criteria, like ipsilateral fornix atrophy, ipsilateral mamillary bodies atrophy, or ipsilateral entorhinal abnormalities. We excluded other MTLE-HS aetiologies like HS due to dual pathology. At the time of the study, 10 patients were not refractory to pharmacological treatment (Table 2). Data concerning FS antecedents was collected from patient medical records and 21 MTLE-HS patients had a history of FS (Table 2). Control individuals were voluntarily recruited among blood donors, ethnically matched, from the same geographic region.

**Table 2:** Clinical and demographic data from MTLE-HS submitted to serum miR-22 determination.

**Clinical/demographic data**	**MTLE-HS (*n* = 40)**	
	**Drug-refractory (*n* = 30)**	**Not refractory (*n* = 10)**
F/M	23 / 17	7 / 3
Age ± SD, years (range)	43.0 ± 12.2 (24–68)	42.4 ± 12.3 (20–60)
Age of onset ± SD, years (range)	12.4 ± 9.8 (1–32)	12.8 ± 11.2 (1–51)
Disease mean duration ± SD, years (range)	30.7 ± 12.6 (6–56)	29.6 ± 13.9 (8–58)
Hippocampal Sclerosis (Left /Right / Bilateral)	18 / 11 / 1	7 / 3 / 0
Febrile seizures antecedents (Yes / No)	15 / 15	6 / 4
AED (0 / 1 / 2 / ≥ 3)	0 / 6 / 8 / 16	1 / 2 / 6 / 1

*AEDs, antiepileptic drugs; F, female; M, male; SD, standard deviation*.

Peripheral blood was collected in tubes without anticoagulant (Vacuette, GBO, Germany), centrifuged at 490 g and serum aliquots were stored at −20°C. RNA was extracted using the miRNeasy^®^ Serum/Plasma Kit (Qiagen, Germany), according to the manufacturer’s instructions. The synthesis of cDNA was performed with the Taqman^®^ MicroRNA reverse Transcription-Applied Biosystems Kit (Applied Biosystems, USA) and a specific primer for miR-22 (Taqman^®^ MicroRNA Assays, Applied Biosystems, USA). The reaction was performed in a BiometraAlfagene thermocycler accordingly to the manufacturer’s instructions. The quantitative RT-PCR amplification was run with specific primers and probes for miR-22 (Taqman^®^ MicroRNA Assays, Applied Biosystems, USA) and NzySpeedy qPCR mastermix (Nzytech, Portugal) in a Corbett Rotor Gene 600 Real Time Thermocycler (Corbett Research, UK). Each reaction was performed in triplicate and the average Ct value was used in the analysis. The relative expression was calculated using the 2^−ΔΔCT^ method. MiR-22 level was evaluated in serum, a cell-free body fluid that is not known to have constant levels of a particular RNA species, hindering expression normalization by an endogenous control or housekeeping gene. To overcome this problem, the same serum volume was used for each subject and the same threshold was used so that expression levels could be compared between samples. Therefore, micro-RNA levels are expressed as 50-Ct (Wang et al., 2010).

### Data Presentation and Statistical Analysis

P2X7R density was expressed as fold change of control individuals. Results are expressed as mean ± standard deviation (SD); “n” shown in graphs represents the total number of individuals. Normal distribution was evaluated with Kolmogorov-Smirnov test. Spearman’s correlation coefficients were used to test interactions between age, age at onset, disease duration, and expression levels. Receiver Operator Characteristic (ROC) analysis was performed to investigate the ability of miR-22 serum levels to discriminate MTLE-HS patients, namely poorer responder to AEDs, from the control population. Statistical analysis was carried out using GraphPad Prism 9 software (La Jolla, CA, USA). Differences in ΔCt were evaluated using the unpaired Student’s *t-*test with Welch correction. For multiple comparisons, one-way ANOVA with Dunnett’s multiple comparison test or two-way ANOVA followed by Holm-Šídák’s multiple comparison test was used, when indicated. *p* < 0.05 values were accepted as significant.

## Results

**Figure 1:**
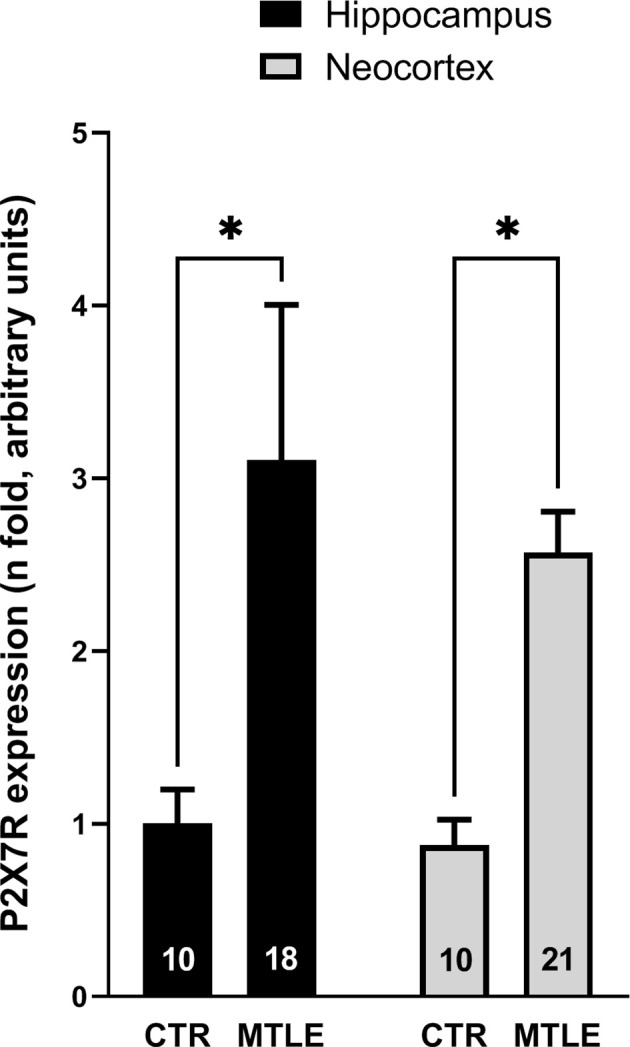
The P2X7R mRNA is overexpressed in the hippocampus and temporal neocortex of drug-refractory MTLE-HS patients submitted to amygdalohippocampectomy compared to non-epileptic cadaveric controls (CTR). qPCR data are expressed as mean ± SD; *n* numbers inside each bar represent the number of individuals among 10 controls and 23 MTLE-HS patients in which quality assessment of retrieved RNA samples was suitable for quantification. ^*^p < 0.05 (two-way ANOVA followed by Holm-Šídák’s multiple comparisons test was used) represents significant differences when compared to control individuals.

**Figure 2:**
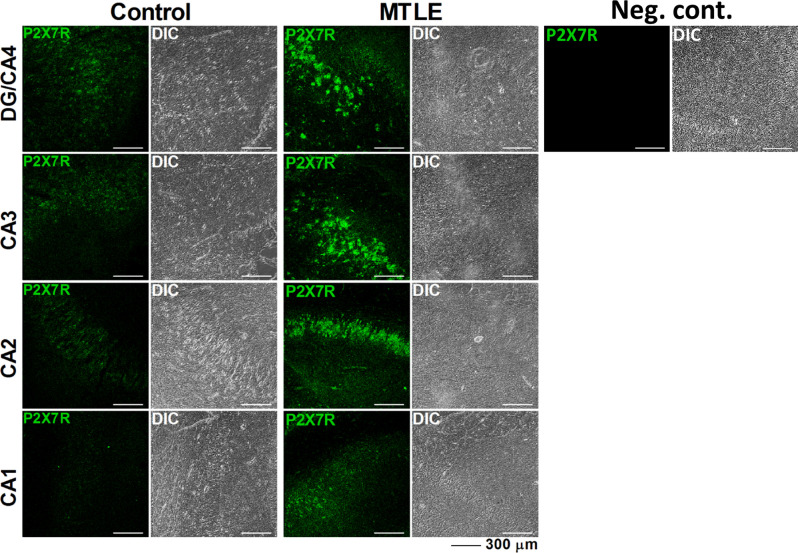
Representative confocal micrographs of different sub-regions of the human hippocampus showing that the P2X7R immunoreactivity (green) is higher in the hippocampus of MTLE-HS patients than of non-epileptic cadaveric controls. A negative control resulting from incubation of the DG/CA4 hippocampal region of an MTLE-HS patient with the anti-rabbit secondary antibody without previous addition of the rabbit anti-P2X7R primary antibody (#APR-004) and differential interference contrast (DIC) images are shown for comparison; three confocal micrographs were obtained per individual; three individuals from each group (control and MTLE-HS) were analyzed showing similar results; scale bars = 300 μm.

P2X7R mRNA transcripts are overexpressed in the hippocampus and temporal neocortex of MTLE-HS patients compared to that observed in control individuals (Figure 1). This was also observed at the P2X7R protein level in all sub-regions of the hippocampus, as demonstrated by immunofluorescence confocal microscopy (Figure 2). Increased P2X7R mRNA amounts in the hippocampus and temporal neocortex were not significantly (*p* > 0.05) correlated with gender, age of epilepsy onset, age at surgery, nor the duration of the disease condition (Table 3). Likewise, no significant differences (*p* > 0.05) were observed in the amount of P2X7R mRNA transcripts measured in the hippocampus and temporal neocortex among MTLE-HS patients’ subgroups taking two, three, or four AED combinations at the time of surgery (data not shown). We also failed to detect differences (*p* > 0.05) in the amounts of P2X7R mRNA transcripts expressed in the hippocampus and temporal neocortex of MTLE-HS patients grouped by the three most prescribed AEDs, carbamazepine (*n* = 12), clonazepam (*n* = 11), and levetiracetam (*n* = 8), to ensure minimal statistical power.

**Table 3:** Spearman’s correlation analysis regarding the P2X7R expression.

	**Age of onset**	**Disease duration**	**P2X7R Hip**	**P2X7R Ctx**
Age of onset				
Correlation Coefficient	1	−0.311	0.326	−0.300
Sig (2-tailed)	- - -	0.148	0.187	0.186
Disease Duration				
Correlation Coefficient	−0.311	1	−0.017	−0.118
Sig (2-tailed)	0.148	- - -	0.948	0.609
P2X7R Hippocampus				
Correlation Coefficient	0.326	−0.017	1	0.383
Sig (2-tailed)	0.187	0.948	- - -	0.053
P2X7R Cortex				
Correlation Coefficient	−0.300	−0.118	0.383	1
Sig (2-tailed)	0.186	0.609	0.053	- - -

**Figure 3:**
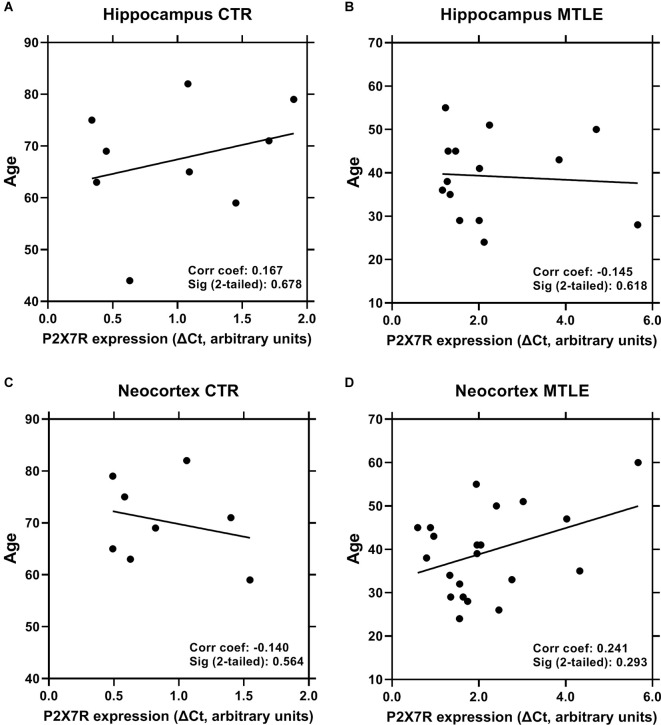
No significant correlation was observed between the P2X7R expression and the age of epileptic patients (panels **B** and **D**) and non-epileptic cadaveric controls (panels **A** and **C**), both in the hippocampus (panels **A** and **B**) and in the temporal neocortex (panels **C** and **D**). Each point represents an individual sample among 10 controls and 23 MTLE-HS patients in which quality assessment of retrieved RNA samples was suitable for quantification. Spearman’s correlation coefficients and significance *p* values (two-tailed) are shown inside each graph.

Considering that epileptic patients submitted to surgery were significantly younger (39.6 ± 9.8 years old, *n* = 23) than the cadaveric controls (67.0 ± 10.9 years old, *n* = 10), and because aging can affect the expression of inflammasome components (Mawhinney et al., 2011), we asked whether age differences could account for increases in the P2X7R expression in the brain of epileptic patients. Using a Spearman’s correlation analysis, we detected no significant correlation between the P2X7R expression and age of epileptic patients (Figures 3B,D), as well as of cadaveric controls (Figures 3A,C), both in the hippocampus (Figures 3A,B) and in the temporal neocortex (Figures 3C,D).

**Figure 4:**
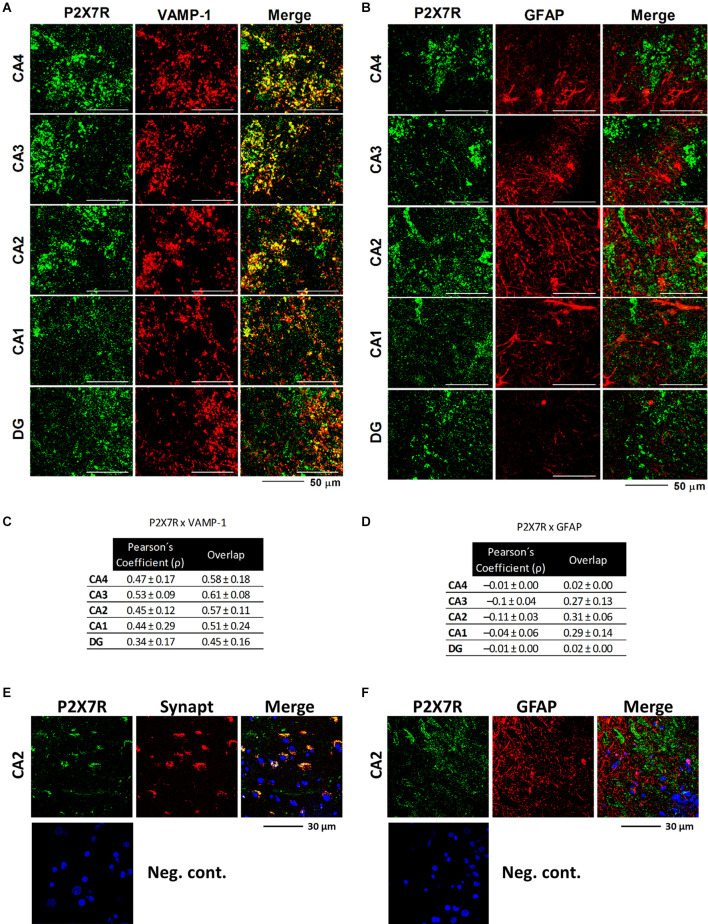
Representative confocal micrographs showing that the P2X7R immunoreactivity is located predominantly in nerve terminals, but not in glial cells, of all sub-regions of the hippocampus of MTLE-HS patients. Synaptic nerve terminals were identified with an antibody against the vesicle-associated membrane protein 1 (VAMP-1 or synaptobrevin 1), whereas astrocytes were stained with an antibody against the glial fibrillary acidic protein (GFAP). Note that VAMP-1-positive nerve terminals (red) are endowed with the P2X7R (green; panel **A**), but no significant co-localization was observed between P2X7R (green) and GFAP (red; panel **B**); scale bars = 50 μm. Data in panels **(C)** and **(D)** correspond to staining overlap and Pearson’s Coefficient (ρ) parameters calculated from three to four confocal micrographs per individual; at least three individuals from each group, control, and MTLE-HS, were analyzed. These parameters were automatically calculated per image with Olympus Fluoview 4.2 Software (Olympus FV1000, Tokyo, Japan) and were used to estimate the co-localization of P2X7R and type-specific cell markers (yellow staining). Overlap between two colors gives values between +1 (total overlap) and 0 (no overlap); the Pearson’s Coefficient (ρ) is a measure of the linear correlation between two variables (stainings), giving values between +1 and −1 inclusive, where 1 is total positive correlation, 0 is no correlation, and −1 is total negative correlation. Higher magnification images show that the P2X7R immunoreactivity also co-localizes with the synaptic vesicle glicoprotein synaptophysin (Synapt), which is one of the most commonly used neuronal cell markers in neuropathology (Panel **E**), but not with GFAP (Panel **F**). Nuclei are stained with DAPI; cross-reactivity for the secondary antibodies was tested in control experiments in which primary antibodies were omitted (negative controls).

The confocal micrographs depicted in Figure 4A show that the P2X7R is predominantly overexpressed in VAMP-1-positive synaptic nerve terminals of all sub-regions of the epileptic hippocampus. Higher magnification images also show that the P2X7R co-localizes with the synaptic vesicle glicoprotein synaptophysin (Synapt), which is one of the most commonly used neuronal cell markers in neuropathology (Figure 4E). The P2X7R did not (*p* > 0.05) co-localize with the astrocytic cell marker, GFAP, despite the extensive astrogliosis existing in this epileptic brain area (Figure 4B,F; higher magnification images are shown in Figure 4F). Co-localization was assessed by evaluating the Pearson’s coefficient (ρ) and staining overlap obtained by merging the two fluorescent channels (yellow staining; Figures 4C,D), as reported in a previous article (Barros-Barbosa et al., 2016).

**Figure 5:**
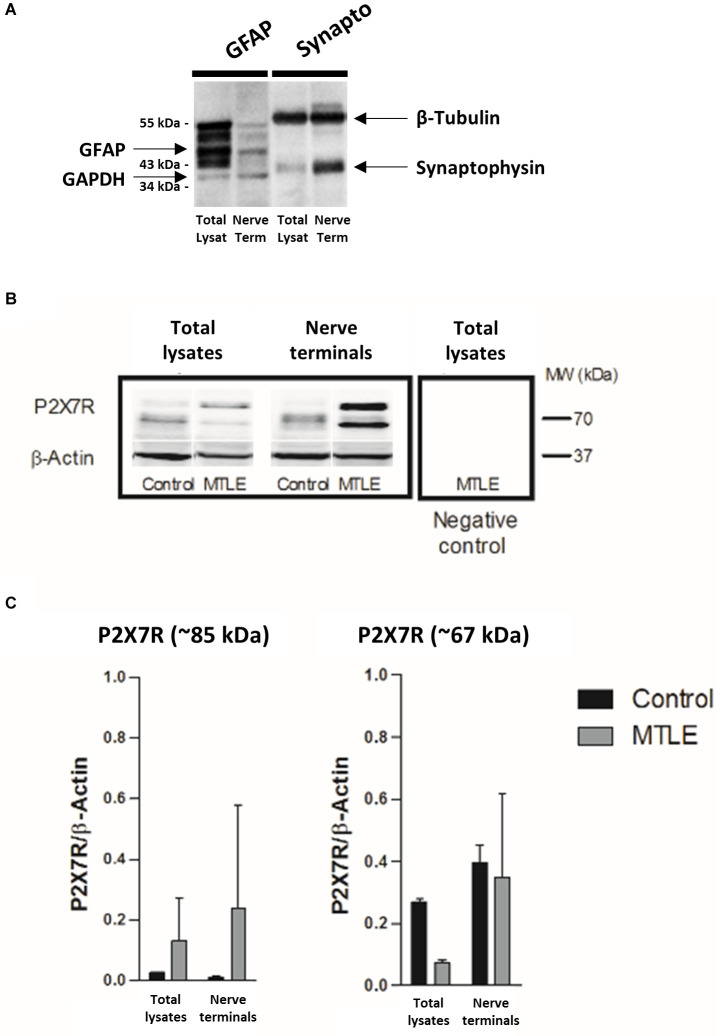
The P2X7R protein is upregulated in nerve terminals of the hippocampus of drug-refractory MTLE-HS human patients. Panel **(A)** shows that using our methodology nerve terminals isolated from the hippocampus of MTLE-HS patients exhibit a higher density of the synaptic vesicle marker, synaptophysin (~34 kDa), compared to the astrocytic cell marker, GFAP, whilst the opposite was observed in total hippocampal lysates. In panel **(B)** are shown representative Western blots of the P2X7R immunoreactivity in total lysates and nerve terminals isolated from the human hippocampus of control individuals and MTLE-HS patients; gels were loaded with 100 μg of protein. Two protein species were recognized by the P2X7R antibody from Alomone (#APR-004, Jerusalem, Israel) corresponding to the naturally occurring 67 kDa receptor isoform and to a higher molecular mass (~85 kDa) P2X7R isotype; the latter is highly enriched in nerve terminals of the hippocampus of MTLE-HS patients compared to non-epileptic controls. Please note that the two bands corresponding to the P2X7R protein disappeared after pre-adsorption of the primary antibody with a control antigen peptide equivalent to the amino-acid residues 576–595 of the intracellular C-terminus of the P2X7R (negative control); β-Actin (38–41 kDa) was used as a reference protein. Panel **(C)** shows computed data obtained from immunoblot experiments; data are expressed as mean ± SD; each individual sample was processed in duplicate; at least three individuals from each group (control and MTLE-HS) were analyzed.

Moreover, we show here that the P2X7R protein is enriched in isolated nerve terminals (Figure 5A) compared to total lysates of the hippocampus of MTLE-HS patients (Figure 5B), which is in agreement with that found in the human temporal neocortex (Jimenez-Pacheco et al., 2013; Barros-Barbosa et al., 2016). While the naturally occurring 67 kDa P2X7R protein predominates in the brain of non-epileptic controls (Figures 5B,C), a higher molecular weight (~85 kDa) protein accounts more for the P2X7R overexpression in epileptic hippocampi (see e.g., Jimenez-Mateos et al., 2019; Mccarthy et al., 2019). Both P2X7R protein isotypes disappeared after pre-adsorption of the membranes with the control antigen peptide corresponding to amino acid residues 576–595 of the P2X7R intracellular C-terminus (Figure 5B, last lane).

**Figure 6:**
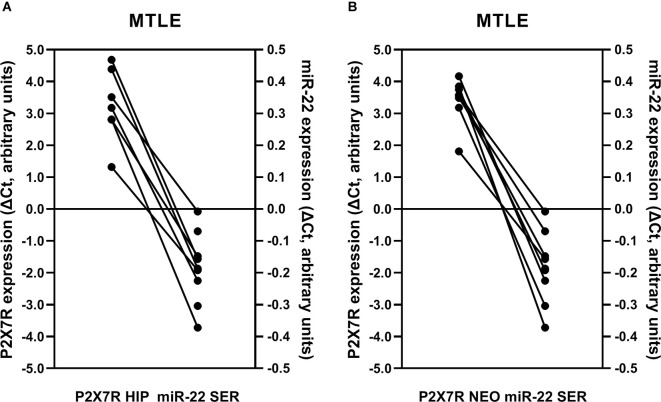
The inverse relationship between miR-22 serum levels and the P2X7R expression in the hippocampus **(A)** and temporal neocortex **(B)** from drug-refractory MTLE-HS patients submitted to amygdalohippocampectomy. Ordinates represent ΔCt variation of P2X7R and miR-22 expression in epileptic patients compared to the corresponding median of non-epileptic controls; 0 represents null variation; positive and negative values correspond to increases and decreases relative to the control population, respectively. Please note that there is a mismatch between miR-22 serum quantifications and P2X7R mRNA determinations in the hippocampus (**A**, two patients missing) and temporal neocortex (**B**, one patient missing) because RNA samples were insufficient or did not pass the quality assessment.

Previous studies conducted in animal models suggest that miR-22 exerts a neuroprotective role in traumatic (Ma et al., 2016) and epileptic (Jimenez-Mateos et al., 2015) brain injuries by a mechanism tightly linked to P2X7R deactivation. Moreover, downregulation of circulating miR-22 was detected following *status epilepticus* in mice (Jimenez-Mateos et al., 2015). This prompted us to investigate if P2X7R overexpression in the hippocampus and temporal neocortex of drug-refractory MTLE-HS patients was associated with the downregulation of miR-22 serum amounts in these patients. An inverse relationship between miR-22 in the serum and the P2X7R mRNA expression in the hippocampus (Figure 6A) and temporal neocortex (Figure 6B) of epileptic patients was verified, i.e., low miR-22 in the serum corresponds to increases in P2X7R gene transcription in these two brain regions.

**Figure 7:**
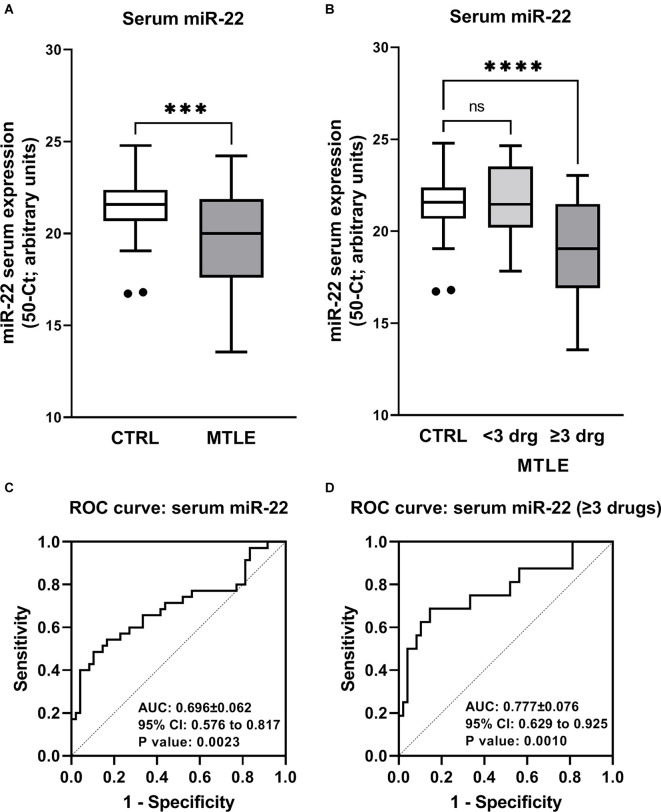
Patients with drug-refractory MTLE-HS exhibit lower miR-22 serum levels than the control population (panel **A**). This difference is exaggerated when patients with poor response to medication (requirement of three or more AEDs) are considered (panel **B**). Boxes and whiskers (Tukey method) represent pooled data from 48 blood donor controls (CTRL) and 40 MTLE-HS patients (panel **A**); in panel **(B)** epileptic patients were subdivided into drug-resistant (≥3 AE Drugs) and non-drug-resistant (<3 AE Drugs). The values plotted individually are outliers according to the Grubbs test. In panel **(A)**, ^***^p < 0.001 (unpaired Student’s *t-*test with Welch correction) represent significant differences compared to the control population; in panel **(B)**, ^****^p < 0.0001 (one-way ANOVA with Dunnett’s multiple comparison test) represent significant differences compared to the control population. ns = non-significant. In panels **(C)** and **(D)**, represented are the receiver-operator characteristics (ROC) curves of miR-22 serum levels considering the whole MTLE-HS patients’ population (panel **C**) or only patients from this cohort taking more than three AEDs (panel **D**). Please note that the area under de curve (AUC) increases considering MTLE-HS patients with poorer response to medication.

As a proof of concept, we also compared the miR-22 serum levels of 40 MTLE-HS patients (23 F/17 M; 43.0 ± 12.2 years old) followed at the Epilepsy Outpatient Clinic of HSA-CHUP with 48 age- and gender-matched blood donor controls (28 F/20 M; 42.0 ± 10.8 years old) with no known neurological disease condition. Figure 7A shows that MTLE-HS patients exhibit significantly lower (*p* = 0.001) miR-22 serum levels than the control population. This difference is even more evident in the subpopulation of epileptic patients requiring more than three different AEDs to control seizures (*p* < 0.001; Figure 7B). Like that observed concerning the P2X7R mRNA expression in the brain, no significant correlations (*p* > 0.05) were observed between mir-22 serum levels and gender, age of onset, age at surgery, and/or duration of the disease condition (Table 4).

**Table 4:** Spearman’s correlation analysis for miR-22 serum expression.

	**Age of onset**	**Disease duration**	**Circulating miR-22**
Age of onset			
Correlation Coefficient	1	−0.89	0.207
Sig (2-tailed)	- - -	0.071	0.200
Disease Duration			
Correlation Coefficient	−0.89	1	0.269
Sig (2-tailed)	0.071	- - -	0.094

The Receiver Operating Characteristics (ROC) graphs are commonly used in medical decision making. ROC graphs have long been used in signal detection theory to depict the trade-off between hit rates and false alarm rates of classifiers. A high area under the ROC curve (0.696; 95% CI 0.576–0.817; *p* = 0.0023) is consistent with low miR-22 serum levels being a sensitive biomarker for discriminating MTLE-HS patients (Figure 7C). The ROC curve analysis with an AUC of 0.777 (95% CI 0.629–0.925; *p* = 0.001) demonstrates that miR-22 serum expression levels (50-Ct) below 20 are highly specific (89%) and sensitive (69%) for discriminating healthy controls from poorer responders to anti-epileptic medication, i.e., MTLE-HS patients requiring more than three AEDs to control seizures (Figure 7D).

## Discussion

Our results show for the first time, that downregulation of miR-22 gene transcripts in the serum is inversely related to P2X7R overexpression in the hippocampus and temporal neocortex of human MTLE-HS patients, which is in agreement with the prediction made in the experimental TLE animal model (Jimenez-Mateos et al., 2015). Data also suggests that the inverse relationship between miR-22 and P2X7R gene transcription may occur soon after the epileptogenic trigger, but once established it is unlikely to be affected by the duration of the disease condition, as well as by the age of onset and gender. A high area under the ROC curve indicates that low miR-22 serum levels may be a highly specific biomarker to anticipate MTLE-HS patients with poor response to medication.

Brain damage and other stressful conditions, such as ischemia, trauma, or excessive neuronal activity, lead to a rapid upregulation of the P2X7R gene transcription and protein translation (North, 2002). This typically coincides with increases in extracellular ATP levels, thus favoring activation of low-affinity slow-desensitizing P2X7 receptors. Signaling through these receptors may affect both neuroinflammation and neurotransmission, which is associated with exacerbation and propagation of seizures throughout the brain. Upregulation of the P2X7R subtype in the temporal neocortex adjacent to the hippocampus, a region that is highly committed to seizure propagation, strengthens the overall pro-epileptic role of this ATP-sensitive receptor subtype predicted by several authors.

Controversy exists regarding the cellular localization of P2X7R in the brain. Previous studies showed that P2X7R are predominantly located on glial cells, including microglia, oligodendrocytes, and astrocytes, although neuronal expression has also been described (Engel et al., 2012b; Barros-Barbosa et al., 2016). Differential cellular expression of the P2X7R seems to be highly dependent on the model and/or stage of the epileptic process under evaluation. For instance, in animal models of TLE or *status epilepticus* (Rappold et al., 2006; Dona et al., 2009; Jimenez-Pacheco et al., 2016), the P2X7R localizes predominantly in microglial cells of the CA3 hippocampal region, thus coinciding with major neuronal cell losses in this brain area (Engel et al., 2012a; Jimenez-Pacheco et al., 2013, 2016; Barros-Barbosa et al., 2016). This pattern dramatically changes in the CA1 and dentate gyrus regions where the P2X7R was found to be more abundant in neuronal cell populations (Dona et al., 2009; Engel et al., 2012a; Jimenez-Mateos et al., 2015). One may also argue that this may reflect different roles of the P2X7R according to distinct phases of epileptogenesis. *Ab initio*, the P2X7R upsurge may be relevant in order to negatively compensate for neuronal overexcitation (Armstrong et al., 2002) and to overcome cellular damage arising from excessive neuronal activity. As a consequence of this phenomenon, there is an increase in extracellular ATP levels with subsequent P2X7R overactivation, promoting proliferation and activation of microglial cells (Monif et al., 2009). This results in synchronous release of pro-inflammatory cytokines (Ferrari et al., 1997; Skaper et al., 2010; Choi et al., 2012), which are required for damaged cell repair. *In vitro* studies suggest the existence of a positive feedback loop between IL-1β and P2X7R (Narcisse et al., 2005), which may explain overexpression of both gene transcripts in the hippocampus and temporal neocortex of this MTLE-HS patients cohort (this study; see also Leal et al., 2017). In this situation of altered excitability, inflammatory microenvironment, and activated microglia, the extracellular levels of ATP continue to escalate and long-term P2X7R activation may lead to sustained and uncontrolled inflammatory reactions, cell death, and hyperexcitability (Sperlagh and Illes, 2014). Prolonged P2X7R activation rends cells more susceptible to ATP-induced death due to the formation of a plasma membrane pores resulting in intracellular Ca^2+^ overload and to the efflux of potassium and other relevant metabolites in a manner that is independent of inflammatory actions (Balosso et al., 2008; Skaper, 2011; Henshall, 2013). This scenario triggers synaptic neurotransmission deficiencies by interfering with glutamate homeostasis and ATP/adenosine metabolism (Barros-Barbosa et al., 2016, 2018). In addition, inflammatory cytokines may also interfere with neurotransmitter signaling, namely *via* NMDA receptors, resulting in neuronal hyperexcitability (Balosso et al., 2008). All these events aggravate neuronal network instability and dysregulation characterizing epilepsy, thus contributing to seizures prolongation and propagation to other brain regions.

Using confocal microscopy for P2X7R protein co-localization with cell-type specific fluorescent markers (VAMP-1 and GFAP for nerve terminals and astrocytes, respectively), together with Western blot analysis to distinguish the P2X7R protein density in isolated nerve terminals *vis-a-vis* total cell membrane lysates, we show here for the first time that P2X7R is predominantly expressed in nerve terminals of the hippocampus of MTLE-HS human patients. Our findings agree with data obtained in experimental TLE animal models, as well as in the temporal neocortex of epileptic patients (Armstrong et al., 2002; Vianna et al., 2002; Yu et al., 2008; Engel et al., 2012a; Jimenez-Pacheco et al., 2013, 2016; Barros-Barbosa et al., 2016). Mounting evidence suggests that neuronal P2X7R activation regulates the extracellular levels of GABA and glutamate (Khakh et al., 2003), indirectly by interfering with neuronal excitability, or more directly due to massive dysregulation of cytoplasmic ion homeostasis and consequent alteration of depolarization thresholds (Armstrong et al., 2002; Sperlagh et al., 2002). In this regard, our group showed that P2X7R activation downmodulates Na^+^-dependent GABA and glutamate uptake in isolated nerve terminals from epileptic patients also contributing to the extracellular accumulation of these neurotransmitters (Barros-Barbosa et al., 2016). It seems that the P2X7R differentially regulates GABA and glutamate release depending on neuronal activity and microenvironment conditions (Barros-Barbosa et al., 2018).

Extracellular GABA accumulation caused by P2X7R over activation might not be protective as one would initially predict, since it may contribute to neuronal hyperexcitability due to paradoxical GABAergic “rundown” in the epileptic human brain (Barros-Barbosa et al., 2016). In line with these observations, it has been shown that overexpression of the P2X7R reduces responsiveness to anti-convulsants that may be overcome by P2X7R antagonists in animal models of *status epilepticus* (Beamer et al., 2021). Some authors claim that there is a reduction in seizure severity associated with reduced neuronal damage (Jimenez-Pacheco et al., 2013; Mesuret et al., 2014), while others argue that only the number and frequency of seizures are reduced (Jimenez-Pacheco et al., 2016). An interesting, yet puzzling, result is that the effect is maintained and sometimes even amplified when treatment with P2X7R antagonists is discontinued (Jimenez-Pacheco et al., 2016). Moreover, P2X7R antagonists have been shown to potentiate the anticonvulsant effects of lorazepam (Engel et al., 2012a) and carbamazepine (Fischer et al., 2016). Notwithstanding this, contradictory results regarding the P2X7R effect in seizure development have been reported using different animal models. For instance, while P2X7R inhibition leads to exacerbation of pilocarpine-induced chronic seizures, a situation that is commonly associated with high neuronal death and reduced astrocytic cell damage in the CA3 hippocampal area, blockage of P2X7R activation had no effect in seizures outcome of kainic acid-induced *status epilepticus* (Kim and Kang, 2011). As mentioned before, these idiosyncrasies may be justified taking into consideration the cellular type that is predominantly affected in each epilepsy model, as well as the mechanism and stage of the epileptogenic process being considered.

Remarkably, two protein species were recognized by the KO-validated P2X7R antibody (Apolloni et al., 2013). One corresponds to the naturally occurring (~67 kDa) P2X7R isotype found in control brain specimens, while the other showing a higher molecular weight (~85 kDa) identified a predominant P2X7R isoform in epileptic hippocampal nerve terminals. The putative clinical significance of the P2X7R enrichment in hippocampal nerve terminals *vis-a-vis* total lysates and the molecular mass gain of this receptor isoform cannot be answered by the present experimental setting, but it certainly deserves to be explored in future functional studies. Epileptogenesis may involve changes in the complex regulatory mechanisms that influence receptors expression, localization, and function, which include epigenetic DNA methylation, alternative splicing, microRNA interference, and post-translational modifications (e.g., phosphorylation, glycosylation, and palmitoylation) affecting receptors trafficking, assembly, and opening (see e.g., Jimenez-Mateos et al., 2019; Mccarthy et al., 2019). Despite AEDs can significantly influence epigenetics, as for instance chromatin-related epigenetic alterations (Navarrete-Modesto et al., 2019), we found any significant correlation between the number and type of combinatorial AED regimens at maximal therapeutic doses and the amount of P2X7R mRNA transcripts detected in the hippocampus and temporal neocortex of drug-refractory MTLE-HS surgical patients.

Mounting evidence suggests that acute and chronic P2X7R expression is tightly controlled in the CNS and that dysregulation of these mechanisms during epileptogenesis may affect disease severity and progression. Unilateral injection of kainic-acid induces P2X7R overexpression in the ipsilateral epileptogenic focus, but not in the contralateral hippocampus (Jimenez-Mateos et al., 2015). This was ascribed to post-transcriptional repression of the P2X7R in the contralateral hippocampus by a microRNA molecule, the miR-22 (Jimenez-Mateos et al., 2015). These authors also showed that miR-22 targeting of the P2X7 purinoceptor opposes seizure development. Coincidently or not, both P2X7R and miR-22 are regulated by the same transcription factor whose action is dependent on intracellular Ca^2+^ levels (Engel et al., 2017). The specificity protein 1 (Sp1) has been shown to induce P2X7R transcription *in vitro* and Sp1 occupancy of the miR-22 promoter region is blocked under conditions of high Ca^2+^ influx into neuronal cells, such as those occurring during excessive P2X7R activation driven by high ATP amounts released from cells during seizures. This calcium-sensitive feed-forward loop regulating the neuronal expression of the ATP-gated P2X7R is accompanied by a pro-convulsive downregulation of miR-22-mediated translational repression of the P2X7R protein synthesis leading to this receptor overexpression at the plasma membrane.

Our results strengthen this theory, since we observed that drug-refractory MTLE-HS patients possess higher than control levels of P2X7R in the hippocampus and adjacent temporal neocortex whilst miR-22 is downregulated in the serum. Our results are also in keeping with those obtained in the brain of epileptic animals used to validate serum microRNA quantification. A similar relationship has also been observed in other neuroinflammatory disease conditions, such as amyotrophic lateral sclerosis (Parisi et al., 2013). One important limitation of our study is that due to technical reasons we were not able to measure miR-22 expression in the brain. For obvious reasons the amount of epileptic brain tissue available for the experiments was very limited, so we were only able to extract total RNA, which prevented the possibility of isolating and accurately quantifying the different RNA subtypes, including non-coding miRs (Brown et al., 2018). Having this in mind and knowing that miRs are very stable in biological fluids where they are thought to reflect remote tissue production (Turchinovich et al., 2012), we believe that serum miR-22 levels may be a clinical surrogate of its production in the brain whose levels may vary inversely to P2X7R expression in the cerebral tissue.

It is particularly interesting to note that MTLE-HS patients controlled with only one or, at least, two AEDs (good responders) had similar miR-22 serum expression levels as control individuals, whereas those requiring more than three AEDs to control seizures (poor responders) typically present low circulating amounts of this microRNA. In an animal model of traumatic brain injury, low miR-22 expression is associated with higher neuronal cell damage (Ma et al., 2016). Recently, it was also shown that miR-22 deficient mice have a more severe epilepsy phenotype, with seizures beginning earlier and being more frequent and longer than in wild-type littermates (Almeida Silva et al., 2020). Such differences are more prominent in early epilepsy stages and these animals overexpress the P2X7R in the brain. Genetic ablation of miR-22 blunted the inflammatory transcriptional response to *status epilepticus* and exacerbated epilepsy highlighting the putative dual role of inflammation in epileptogenesis. While this feature may be detrimental during later epileptogenesis stages when global cell dysregulation is observed, it may be beneficial at the initial inflammatory phase to remove cellular debris and to promote cell repair in order to recover homeostasis. In keeping with this, it has been demonstrated that miR-22 is an important regulator of morphogenesis of newly formed neurons in adults and it plays a role in suppressing aberrant neurogenesis associated with *status epilepticus* (Beamer et al., 2018).

Our results suggest that circulating miR-22 may be a promising biomarker of drug refractoriness in MTLE-HS patients. We are aware that changes in circulating levels of miR-22 may be produced by other (yet to resolve) clinical conditions besides MTLE-HS. Nevertheless, we anticipate that measuring serum miR-22 amounts might be useful if included with other putative biomarkers in epileptic patients’ evaluation. In this regard, data from animal studies also point toward the quantification of P2X7R plasma levels as a useful biomarker for the diagnosis and therapeutic follow-up of epileptic individuals (Conte et al., 2021). Monocytes have been claimed as the main source of circulating PX7R, yet excessive brain production of this protein in epileptics cannot be excluded. Given the inverse relationship between circulating P2X7R and miR-22, it is tempting to speculate that both biomarkers possess the same neuronal origin in epileptics, and this deserves to be elucidated in future studies using larger multi-center cohorts.

Chronic epilepsy patients’ refractory to multiple AED regimens, such as those referred to neurosurgical treatment in the present study, likely present seizures that are more frequent and severe. It is tempting to assume that, in these cases, sustained extracellular ATP accumulation together with the 85 kDa P2X7R protein overexpression, resulting in uncontrolled neuroinflammatory reaction and synaptic transmission dysregulation, may act as a positive feed-forward detrimental loop ending up in cellular damage and unrestrained seizures progression. Given the physiological role of the P2X7R to higher brain functions, like memory and cognition, the purinergic dysregulation may be more severe in predisposed individuals, who also may present more significant memory deficits as a co-morbid clinical condition. As a matter of fact, genetically predisposed individuals of the rs16944TT genotype displaying higher IL-1β (and P2X7) expression levels are more susceptible to developing MTLE-HS and seizure recurrence (Leal et al., 2018).

The mechanisms underlying P2X7R overexpression promoted by de-repression of miR-22-induced post-translational modifications may constitute novel molecular targets for the treatment of drug-refractory MTLE-HS patients. The timing of therapeutic intervention and whether it can effectively revert epileptogenesis and disease progression requires further investigations in animal models. Once proven, candidates for this putative novel therapeutic intervention may be selected by a simple blood test among drug-refractory epileptics exhibiting low miR-22 serum levels.

## Data Availability Statement

The raw data supporting the conclusions of this article will be made available by the authors, without undue reservation.

## Ethics Statement

The studies involving human participants were reviewed and approved by Hospital de Santo António—Centro Hospitalar Universitário do Porto/Instituto de Ciências Biomédicas de Abel Salazar—Universidade do Porto. The patients/participants provided their written informed consent to participate in this study. Brain samples made available by the Instituto Nacional de Medicina Legal e Ciências Forenses—Delegação do Norte (INMLCF-DN) were obtained according to Decree-Law 274/99, of 22 July, published in Diário da República—1st SERIE A, No. 169, of 22-07-1999, Page 4522, regarding the regulation on the ethical use of human cadaveric tissue for research in Portugal.

## Author Contributions

BGL, and PC-S: conceptualization and writing—original draft preparation. BGL, AB-B, FF, MGL, JC, RR, AS, CC, RM-F, RS, JF, JL, and JR: methodology. BGL, AB-B, FF, MGL, JC, RR, and AMS: data curation. BGL, AMS, and PC-S: formal analysis. All authors: writing—review. BGL, JC, AMS, and PC-S: funding aquisition. AMS, PC, and PC-S: supervision. All authors contributed to the article and approved the submitted version.

## Conflict of Interest

The authors declare that the research was conducted in the absence of any commercial or financial relationships that could be construed as a potential conflict of interest.

## Publisher’s Note

All claims expressed in this article are solely those of the authors and do not necessarily represent those of their affiliated organizations, or those of the publisher, the editors and the reviewers. Any product that may be evaluated in this article, or claim that may be made by its manufacturer, is not guaranteed or endorsed by the publisher.
